# Bacterial Toxins Fuel Disease Progression in Cutaneous T-Cell Lymphoma

**DOI:** 10.3390/toxins5081402

**Published:** 2013-08-14

**Authors:** Andreas Willerslev-Olsen, Thorbjørn Krejsgaard, Lise M. Lindahl, Charlotte Menne Bonefeld, Mariusz A. Wasik, Sergei B. Koralov, Carsten Geisler, Mogens Kilian, Lars Iversen, Anders Woetmann, Niels Odum

**Affiliations:** 1 Department of International Health, Immunology and Microbiology, University of Copenhagen, Copenhagen 2200, Denmark; E-Mails: awo@sund.ku.dk (A.W.-O.); thorkr@sund.ku.dk (T.K.); cmenne@sund.ku.dk (C.M.B.); cge@sund.ku.dk (C.G.); awoetmann@sund.ku.dk (A.W.); 2 Department of Dermatology, Aarhus University Hospital, Aarhus 8000, Denmark; E-Mails: lise.lindahl@ki.au.dk (L.M.L.); lars.iversen@ki.au.dk (L.I.); 3 Department of Pathology and Laboratory Medicine, University of Pennsylvania, Philadelphia, PA 19104, USA; E-Mail: wasik@mail.med.upenn.edu; 4 Department of Pathology, NYU Langone Medical Center, New York, NY 10016, USA; E-Mail: sergei.koralov@nyumc.org; 5 Department of Biomedicine, Aarhus University, Aarhus 8000, Denmark; E-Mail: kilian@microbiology.au.dk

**Keywords:** cutaneous T-cell lymphoma, infections, *Staphylococcus aureus*, enterotoxins, superantigens

## Abstract

In patients with cutaneous T-cell lymphoma (CTCL) bacterial infections constitute a major clinical problem caused by compromised skin barrier and a progressive immunodeficiency. Indeed, the majority of patients with advanced disease die from infections with bacteria, e.g., *Staphylococcus aureus*. Bacterial toxins such as staphylococcal enterotoxins (SE) have long been suspected to be involved in the pathogenesis in CTCL. Here, we review links between bacterial infections and CTCL with focus on earlier studies addressing a direct role of SE on malignant T cells and recent data indicating novel indirect mechanisms involving SE- and cytokine-driven cross-talk between malignant- and non-malignant T cells.

## 1. Introduction

Cutaneous T-cell lymphoma (CTCL) comprises a heterogeneous group of lymphoproliferative disorders defined by primary expansion of malignant T lymphocytes in the skin. The two most common forms, mycosis fungoides (MF) and Sézary syndrome (SS), constitute approximately 50%–70% of all *de novo* cases of CTCL, with MF accounting for the majority of cases. In this review, CTCL will refer exclusively to mycosis fungoides and Sézary syndrome. Early skin lesions in CTCL usually present as erythematous patches that notoriously resemble benign inflammatory skin disorders like psoriasis, chronic eczema or atopic dermatitis—collectively, making an early diagnosis very difficult [1,2,3,4,5,6] even though promising new approaches using miRNA expression profiling seem to discriminate between the benign inflammatory and malignant conditions inflammation with high accuracy [7,8]. Although patients diagnosed in the early stages of disease often experience an indolent disease course, a subgroup of patients experience an aggressive clinical course with tumor development, skin ulceration, involvement of lymph nodes, bone marrow and internal organs and gradual development of immunodeficiency at later stages of disease. Concomitant with disease progression there is a decrease in normal lymphocyte count and functionality and, consequently, advanced disease may be associated with profound immune deregulation [1,2,9,10]. The etiology of CTCL has long puzzled researchers and a wide range of risk factors has been examined in this regard. Chromosomal instability and abnormal expression of genes involved in cell cycle control and proliferation has been reported several times in CTCL studies [11,12,13]. However, in contrast to other hematological disorders, in CTCL well documented etiological or predisposing genetic factors remain elusive. Occupational and environmental factors have been proposed in some studies but with limited reproducibility and a lack of any evident biological causality [14,15,16]. Yet, a recent finding by Duvic and colleagues sheds light on a previously suspected link between drugs (thiazide used in the treatment of hypertension) and CTCL [17] indicating that environmental factors might indeed play a role in a subset of patients with chemical or biological agents acting as inciting agents in the context of this T cell lymphoma. Familial aggregation of CTCL incidences has been described [18] and a correlation between CTCL disease occurrence and certain human leukocyte antigen (HLA) alleles has also been observed [19]. 

## 2. High Prevalence of Infections

High incidence of infections is a common clinical experience in CTCL [20,21,22]. Axelrod *et al.* examined and quantified different types of infection in 356 CTCL patients [21]. Among the 478 documented infections, 396 were of bacterial origin with the remaining identified as viral, fungal or parasitic. Their study documented that skin was by far the most prevalent site of infection and that risk of infection was intimately associated with the disease stage. Thus, these findings supported the clinical experience that major morbidity and mortality stems from infections and also that patients with progressive disease die more frequently from infection rather than from the CTCL *per se* [21,23]. These important findings prompt the question whether the high incidence of infections in CTCL patients is a mere consequence of a compromised skin barrier, a suppressed immune system, or a combination of both.

## 3. Immunopathogenesis

CTCL progression is typically associated with immune suppression. The malignant cells normally exhibit a mature memory CD4 T cell phenotype and express a range of skin-homing receptors in the initial disease stages, which contribute to the characteristic epidermotropism of malignant T cells [6,10].

**Figure 1:**
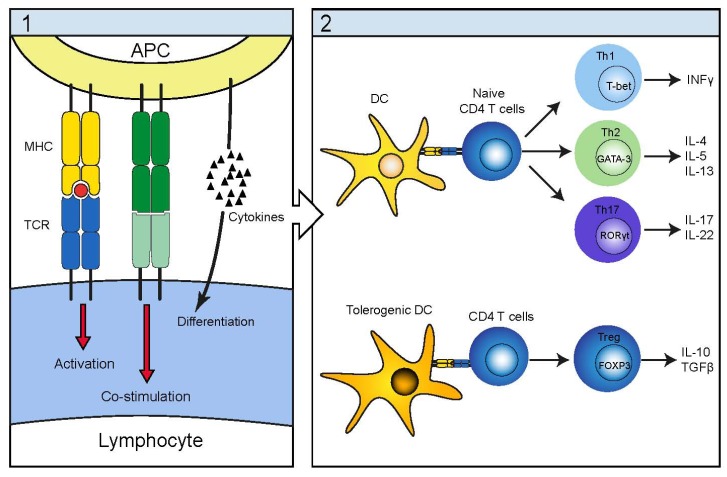
Schematic illustration of the antigen presenting cells (APC) antigen presentation and cytokine release together with the subsequent induction of different lymphocyte helper subsets. (**1**) The APC delivers three signals required for successful lymphocyte activation; antigen presentation, co-stimulation and cytokine release with cytokines being the major determinant of lymphocyte subset induction; (**2**) Additionally dendritic cells DC are able to induce a regulatory phenotype either by the absence of co-stimulation (immature DC’s lack CD80/86) or by activation of lymphocytes in a regulatory cytokine environment (tolerogenic DC’s).

The immunopathogenesis in CTCL is characterized by a gradual shift of cytokine profile in lesional tissue. Early lesions contain a large proportion of non-malignant cells, which primarily consist of dendritic cells, macrophages and tumor-infiltrating cytotoxic CD8 and CD4 T cells [6,10,24]. CD4 T cells may display several different phenotypes depending on their specific activation as illustrated in Figure 1. While the CD4 T cell helper type 1 (T_H_1) is crucial in promoting an effective cellular immune response and as such beneficial in an anti-tumor response, the T_H_2 phenotype is on the contrary promoting a humoral immune response. The more recently recognized T_H_17 cell is believed to be important in certain microbial infection while the T regulatory phenotype is paramount in establishing and sustaining peripheral tolerance. 

In CTCL, the early infiltrating CD4 T cells display a T_H_1 phenotype and in concert, these immune cells are seemingly capable of controlling CTCL cell expansion via cytokines and cytotoxicity [25,26,27,28]. Accordingly, it has been shown that the presence of cytotoxic CD8 T cells within the CTCL lesions is a positive prognostic factor, and several case reports have evidenced that use of the immunosuppressant cyclosporine in treatment of CTCL accelerates disease progression and large cell transformation [10,29,30]. During the disease progression, the concentration of T_H_1 cytokines decreases in contrast to an increased production of T_H_2 cytokines and angiogenetic and lymphangiogenetic factors such as VEGF-A and VEGF-C [10,31,32,33,34,35]. This increasing bias towards a T_H_2 immune response obstructs an effective cellular immune response and can be framed within the immunoediting hypothesis as a process in which the malignancy transitions from an equilibrium phase to a tumor escape phase. Indeed, as the disease progresses, the malignant T cells display an increased expression of B lymphoid tyrosine kinase (BLK), and cyclooxygenase 2 (COX-2) as well as activation of signal transducers and activators of transcription-3 (STAT3) and STAT5 [36,37,38,39], which in turn drive expression of T_H_2 cytokines, oncomiRs (miR-155), and the suppressor of cytokine signaling 3 (SOCS3) [37,40]. The enhanced expression of SOCS3 has been shown to protect malignant T cells from growth-inhibition by pro-inflammatory cytokines such as interferon-alpha (IFNα) [41]. Because IFNα is used for treatment of CTCL, the development of IFNα resistance comprises a pressing clinical problem [41].

Furthermore, direct diversion of anti-tumor immune response been attributed to the malignant T cells in CTCL. Several studies have demonstrated forkhead box P3 (FOXP3) expression in malignant T cells in a subset of patients [42,43,44,45] and upregulation of cytotoxic T-lymphocyte antigen 4 (CTLA-4) in a stage-dependent manner [46]. Likewise the interaction of programmed death protein 1 (PD-1) and its ligand, PD-L1 has been associated with immune evasion in CTCL [47,48] as these cell surface molecules are involved in the induction and maintenance of peripheral T cell tolerance. The increased expression of PD-L1 on neoplastic T cells has been hypothesized to involve the aberrant and constitutive activation of the janus associated kinase 3 (JAK3)-dependent STAT3 cell signaling pathway which is also allegedly a key player in sustaining tissue inflammation while antagonizing tumor immunity [49]. Furthermore, the constitutively active STAT3 induces the secretion of the two potent immunosuppressants; IL-10 and transforming growth factor-beta (TGFβ) [44,45,50]. Collectively, the expression and secretion of the above mentioned molecules supports the model originally proposed by Berger and colleagues who suggest that CTCL T cells maintain dendritic cell immaturity by the release of regulatory cytokines. Further, according to their hypothesis, this results in polarization of the DC’s towards a tolerogenic phenotype, rather than an activating phenotype. In turn, this subtype of DC should promote malignant T cell proliferation and the acquisition of immunosuppressive charactheristics [50,51]. 

Finally, the immunodeficiency in late stage CTCL could also caused by a gradual displacement of non-malignant T cells by the expanding malignant T cell clones; in other words, that the malignant T cells eventually outcompete and substitute the non-malignant T cell population, which results in a state reminiscent of advanced AIDS with a lack of functional CD4 T helper cells and severe immunosuppression [1,2,10,52]. 

**Figure 2:**
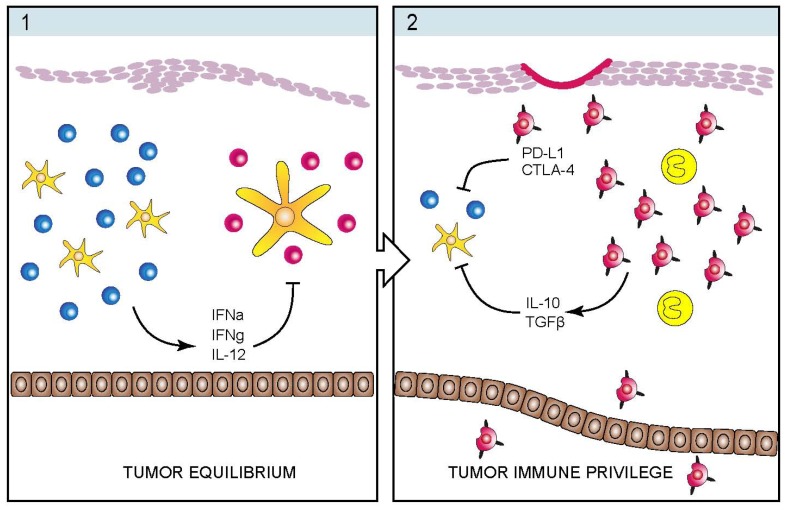
Schematic illustration of the transition from a state of tumor equilibrium to a state of tumor immune privilege. The tumor equilibrium state (**1**) is characterized by T cell- and cytokine-mediated control of tumor progression. Conversely, the state of tumor immune privilege (**2**) is predominated by regulatory signals and cytokines allowing for immune evasion and tumor progression and metastasis. (Yellow: DC; blue: nonmalignant T cell; red: malignant T cell).

Figure 2 shows an illustration of our current view of the dynamic immunological changes during disease progression. Thus, the interplay between malignant T cells, dendritic cells and infiltrating and/or skin-resident, non-malignant T cells change dramatically as the disease progress from an indolent condition to an aggressive cancer. In early stages and non-progressive disease, dendritic cells produce interferon-alpha (IFNa), non-malignant T cells produce T_H_1 cytokines (such as IL-12 and IFNg), and CD8 cytotoxic T cells produce granzymes and mediate direct killing of malignant T cells. These events generate a hostile environment inhibiting malignant proliferation—yet without eradicating the malignant T cell clone—*i.e.*, the tumor lesion is kept in a “state of equilibrium” without expansion and spreading (Figure 2(1)). As malignant T cells change and begin expressing immune modulatory molecules and cytokines (which inhibit the immune control), a “tumor immunological privilege” is established. This “immune privilege” shelter malignant T cells from inhibitory signals allowing for malignant proliferation and induction of immunosuppression and eventually, immunodeficiency (Figure 2(2)).

Malignant T cells in CTCL display a considerable degree of phenotypic heterogeneity, which amongst other things, is believed to impact disease aggressiveness and response to treatment [6,24,53]. Recent studies indicate that this heterogeneity is highly dependent upon crosstalk between malignant T cells and the tumor environment, in that malignant T cells have been shown to secrete a wide array of cytokines, which collectively may activate keratinocytes and surrounding stromal cells and thus sustain tissue inflammation. In return, the activated microenvironment impregnates the malleable malignant T cells with capacities resembling either regulatory T cells or different T helper subsets. Two members of the IL-17 family of cytokines: IL-17A and IL-17F, have recently been implicated in CTCL pathogenesis [54,55,56]. Expression of IL-17A and IL-17F is increased in skin lesions and comparable to the expression levels in lesional psoriatic skin [55]. Noteworthy, heterogeneity in IL-17A and IL-17F expression existed among CTCL patients with some patients having normal or near normal expression whereas others had highly increased levels of IL-17 cytokines. Importantly, an elevated expression of IL-17F correlated with progressive disease [55]. Given the observations of increased IL-17 expression in CTCL patients with bacterial infections [54], we propose a link between bacterial infection, expression of IL-17F and the disease progression. However, it remains to be determined whether IL-17F and other IL-17 family cytokines are fostering disease progression via induction of angiogenesis or other as yet unidentified mechanisms or if an increased expression of these cytokines is a sign of a “frustrated” immune response unable to combat the bacterial infection.

## 4. Infectious Etiology

It has previously been hypothesized that infectious agents (such as a retrovirus) were responsible for the outgrowth of neoplastic T cells and as such are a primary etiological factor in CTCL. MacKie originally launched this hypothesis in 1981 by proposing that CTCL arises from an initial viral infection of epidermal antigen presenting cells [57]. Mackie was inspired by observations of distinctive aggregates of epidermal dendritic cells and T cells in MF patients called Pautrier’s abscesses and also from reports of retrovirus-like particles observed in malignant CTCL T cell cultures [58]. Furthermore, viral antigens have the potential to induce loss of T cell receptor (TCR) diversity, which is a characteristic feature of CTCL [59,60]. This may occur when cross-reactivity exists between viral and auto antigens. According to the hypothesis, autoantigen can sustain proliferation and activation of an autoreactive T cell population following virus eradication thereby resulting in a narrowing of the TCR repertoire [61]. 

The idea of an infectious etiologic agent gained momentum by the earlier discovery of HTLV-1 and its association with adult T cell lymphoma (ATL) [62]. The distinction between the two diseases was not initially recognized due to the clinical, pathological, and histological similarities—although ATL was later established as an unique HTLV-1 induced entity [63]. However, the analogy between CTCL and ATL and the—at the time newly discovered—T-lymphotropic retrovirus, HIV-1 seemed conspicuous [64] and motivated researchers to search for a retroviral culprit in CTCL. Concordantly, retroviral activity and HTLV-like particles in peripheral blood mononuclear cells derived from CTCL patients [65], and successful polymerase chain reaction (PCR) amplification of HTLV-1 sequences was also reported in CTCL skin biopsy specimens [66]. However, controversies arose and later, well-performed, controlled studies failed to associate HTLV-1 with CTCL and the hypothesis of HTLV-1 as the etiological factor in CTCL was put to rest [67,68,69]. Other viruses such as Epstein-Barr, herpes virus 6-8, and cytomegalovirus were later suspected to be involved in CTCL but so far, the associations have been relatively weak and not (yet) convincingly reproduced [70,71,72,73,74].

In addition, bacterial agents have been assigned a direct role in the etiology of CTCL. One candidate was *Chlamydia pneumonia*, which was suggested to foster CTCL through the secretion of a Sézary T cell activating factor (SAF) [75]. Chronic infection with *Chlamydia* was believed to facilitate chronic expansion of *Chlamydia*-specific T cells and the combination of SAF and chronic T cell activation was hypothesized to lead steadily to the development of CTCL [76]. *Borrelia burgdorferi* has also been implicated and in 2006 Bonin and colleagues [77] reported on a minor association between *Borrelia burgdorferi* and CTCL in a population endemic for *Borrelia* infection. Later they suggested that *Borrelia* in conjunction with HTLV-1 (or other infectious agents) can provide a persistent antigenic stimulation, which contributes to the transformation and expansion of T lymphocytes [20]. However, subsequent studies failed to detect a significant presence of *Chlamydia pneumonia* and *Borrelia burgdorferi* in CTCL skin specimens [78,79]. Sporadic case reports [80,81,82] describe other infectious agents of various types but generally they may reflect the findings by Axelrod and colleagues, of a very high degree of diversity in infectious agents present in CTCL patients [21]. As mentioned above, a multitude of pathogens have been isolated from CTCL patients and recurrent infections comprise a large clinical challenge in the care of CTCL patients. The pathogens most frequently associated with CTCL are listed in Table 1, Table 2.

**Table 1:** Prevalence of the most frequent bacterial and viral pathogens associated with cutaneous T-cell lymphoma (CTCL) disease. Patient cohort included 356 CTCL patients. Modified from Axelrod *et al.* 1992 [21].

Bacteria	Number of infections	Frequency
*Staphylococcus aureus*	117	33%–38% *
*Enterobacteriaceae*	38	10.7%
Beta-hemolytic streptococci	35	9.8%
*Pseudomonas aeruginosa*	12	3.4%
Viruses		
Herpes zoster	34	9.6%
Herpes simplex	30	8.4%

* A general study by Axelrod *et al.* (1992) [21] reports that 33% of infections in CTCL are *Staphylococcus aureus*. Jackow *et al.* (1997) [83] detects *Staphylococcus aureus* in 38% of examined CTCL patients.

**Table 2:** Complications associated with infections in CTCL.

Co-morbidity from infections	
Bacterial infections	bacteremia, pneumonia, intra-abdominal infections
Viral infections	ulcerative skin lesions, dissemination (Kaposi varicelliform eruption)

## 5. *Staphylococcus*

*Staphylococcus aureus* is a major source of morbidity in CTCL causing chronic or recurrent skin infections and life-threatening systemic infections such as sepsis, pneumonia and intra-abdominal infections [21,22,23,84]. *S. aureus* is renowned for its ability to produce staphylococcal enterotoxins (SE) (also known as superantigens) [85,86]. Superantigens are characterized by their ability to activate large fractions of T lymphocytes by crosslinking MHC class 2 molecules and T-cell receptors (independently of antigen specificity of the TCR and the antigen-peptide-binding groove in the MHC) thereby circumventing normal antigen processing and presentation. In 1992, Tokura *et al.* showed that malignant CTCL cells responded to SE in a TCR variable β chain (TCRVβ) restricted manner suggesting a possible involvement in the disease [87]. Later, Duvic and colleagues [83] examined 42 CTCL patients with advanced disease (SS or advanced MF with erythrodermia) for bacterial colonization in skin and blood and found that 76% of the patients harbored staphylococci, of which 50% were SE-producing strains of *S. aureus*. Moreover, all patients with toxic shock syndrome toxin-1 (TSST-1)-producing *Staphylococcus aureus* infections had an expansion of TSST-1 specific T cells expressing the appropriate Vβ2 T cell receptors [83]. This observation suggests that superantigens such as SE may be involved in CTCL pathogenesis, as these toxins can facilitate the observed Vβ-restricted T cell expansion [88]. It was hypothesized that SE provide a persistent antigen stimulus for T lymphocytes driving malignant T cell expansion. This notion has been propelled by multiple reports of skewed or diminished T cell receptor repertoire in CTCL patients as discussed below. However, as these studies examined only patients with advanced disease, they actually do not provide evidence for a key role of SE in the etiology and early stage of CTCL. 

## 6. TCRVβ Restriction

By spectratyping the variable regions of the TCR’s β-chain Yawalkar *et al.* [60] demonstrated that half of all early-stage patients and all late-stage patients exhibited a highly diminished complexity of the TCR repertoire compared to the diverse repertoire displayed by normal peripheral T cells [60]. The shrunken TCR repertoire could not reflect a simple monoclonal expansion, as multiple Vβ-families were overrepresented while others were underrepresented or completely absent. In short, Vβ-family distribution failed to follow a normal Gaussian distribution pattern. This skewing of the TCR repertoire was hypothesized to be the result of superantigens such as SE. Superantigens may skew the TCR Vβ repertoire by two mechanisms: 

(1) One involves the previously mentioned direct mechanism by polyclonal activation and proliferation of Vβ-specific T cells following TCR ligation [83,85,86]. Such Vβ-specific expansion by superantigens was suggested by Linneman [89], based on an early-stage CTCL patient, who displayed a dominant Vβ5 T cell population in the skin biopsies. The idea is that superantigen responsive malignant T cells receive activation-signals and thus obtain a growth advantage allowing them to out-compete non-transformed cells. 

(2) The other mechanism involves polyclonal expansion followed by activation-induced cell death of superantigen reactive T cells, which results in a relative expansion of the remaining Vβ-families and thus induces a reciprocal superantigen Vβ-signature. This mechanism has been demonstrated by McCormack and colleagues in a series of mouse studies [90] and subsequently expanded to humans by Vonderheid and colleagues [91] in a cohort of 49 CTCL patients in which a majority of whom exhibited increased Vβ5 usage relative to other Vβ families, usually predominant in normal CD4 T cells. By investigating the TCRα and TCRβ gene rearrangement in 29 CTCL patients Van der Fits [92] concluded that the absence of an unambiguous similarity in the complementarity-determining regions argues against the notion of a single ordinary antigen delivering persistent and pathogenic antigen stimulation in CTCL. However, the skewed Vβ and Jβ gene usage suggested the possibility that also here superantigens may be responsible for the restricted TCR repertoire. It remains to be definitely demonstrated by which mechanism superantigens induce polyclonal T cell proliferation in CTCL. However, since Fas receptor expression has been shown to be effectively down-regulated [25,93,94] and anti-apoptotic pathways such as B-cell lymphoma 2 (Bcl-2) and programmed cell death protein 10 (PDCD10) are enhanced in the CTCL clones [95,96], it is tempting to speculate that malignant T cells can evade Fas induced apoptosis after superantigen activation whereas non-malignant T cells expressing the corresponding Vβ TCR families are deleted. Removal by apoptosis of T_H_1 T cell subsets producing interferon and other inflammatory cytokines, which keep malignant T cells in check, might indirectly promote expansion of malignant T cells.

Although several studies provide circumstantial evidence of superantigen-induced Vβ TCR-associated oligo/poly-clonality in CTCL patients, other groups fail to see “Vβ-signatures” indicative of superantigen involvement. In contrast, these studies observe a monoclonal expansion of malignant T cells [97,98,99,100]. This discrepancy might, amongst other factors, depend in part on the disease-stage of the examined patients, as studies tend to show increasing monoclonality of T cell populations with progression [1,2,4,60]. Indeed, it may also simply rely on the inherent heterogeneity of CTCL; *i.e.*, the disease may in some patients manifest itself as an oligoclonal or skewed polyclonal expansion of T cells while in others it develops as a monoclonal entity. Collectively the above mentioned observations fail to clarify whether infections and infectious superantigens such as SE, function as a primary causative factor—or if they are a secondary event resulting from a weakened immune system and compromised skin barriers. Therefore, further studies are required in order to ascertain whether or not superantigens directly facilitate early expansion of pre-malignant T cells in CTCL, and it is justified to conclude that definitive evidence for an etiological role of superantigens in CTCL is currently still lacking. 

## 7. Indirect Mechanism of Action

**Figure 3:**
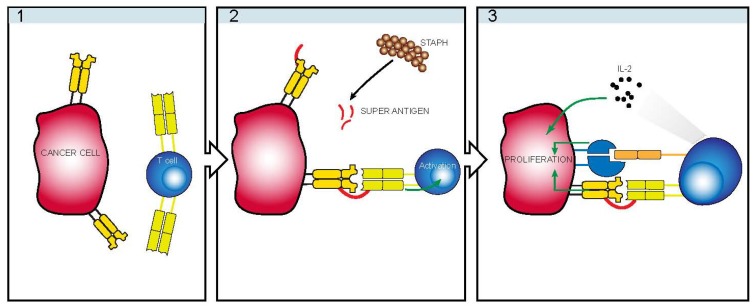
Schematic illustration of SE-mediated cross-talk between malignant and non-malignant T cells. Malignant T cells often display deficient expression and function of the TCR/CD3 complex and may not respond directly to bacterial superantigens such as staphylococcal enterotoxins (SE). Instead, malignant T cells often express MHC class II molecules, which are high-affinity receptors for SE (**1**). Non-malignant T cells with the appropriate Vb TCR respond to SE presented by malignant T cells (**2**, **3**) or by antigen presenting cells (APC) (not shown). SE-mediated cross-talk between malignant and non-malignant T cells triggers cell-to-cell contact and production of growth factors, which in turn promote proliferation of malignant T cells (**3**) [104].

The mechanism of oligoclonal expansion of premalignant T cells, should it occur by superantigen stimulation, is confounded by the fact that several studies of T cells from CTCL patients (including the early patch-plaque stage) show that malignant T cells often display deficient function and/or deficient expression of CD3 TCR complex [101,102,103]. Recently, our group has proposed a novel role of bacterial toxins in disease progression [104]. As illustrated in Figure 2, we observed that whereas malignant T cells did not respond directly to bacterial superantigens, they proliferated vigorously in response to SE in co-cultures with non-malignant T cells indicating an indirect mechanism for growth promotion by SE [104]. Noteworthy, malignant T cells often express major histocompatibility complex (MHC) class II molecules [104], which are high-affinity ligands for bacterial toxins such as SE [105]. Thus, even with defective TCR/CD3 complex, malignant T cells are able to bind SE (Figure 3(2)) and stimulate non-malignant T cells to produce growth factors such as interleukin-2 (IL-2), which in turn promotes growth of the malignant T cells (Figure 3(3)). In addition, toxin-induced cell-to-cell contact between malignant and non-malignant T cells also triggers growth-promoting signals via lymphocyte function associated antigen 3 (LFA-3)/CD2- dependent mechanism (Figure 3(3)) [104]. Importantly, both growth factor and cell-to-cell contact dependent growth stimulation of malignant T cells require MHC class II ligation by the bacterial toxin and expression of a functional TCR/CD3 complex by the non-malignant T cells [104]. In this regard, it is worth mentioning that MHC class II ligation by SE enhances cell-to-cell adhesion [106,107] and IL-2-induced T cell proliferation through an increased activation of ZAP70/p72syk, PLCg1, and expression of the IL-2RA subunit [108,109,110,111,112,113]. Combined, these findings suggest a novel mechanism of tumor growth promotion by SE involving an indirect stimulation of malignant cell proliferation involving LFA-3/CD2-mediated cell-cell contact and soluble growth factors such as IL-2 and other as yet unidentified factors provided by the toxins-activated non-malignant T cells [104]. 

If this mechanism is operating in patients, it indicates that bacterial infections, and especially infections with superantigen-producing bacteria, indirectly activate malignant T cells through help from non-malignant T cells. The activation is not restricted by the Vβ-family on the malignant T cells, but only by their expression of MHC class II molecules or MHC class II expression by other surrounding cells types and their presentation of superantigens to Vβ-specific non-malignant T cells. The ability of non-malignant T cells to inadvertently promote tumor expansion is not unique to CTCL and has previously been reported in other malignancies. Specifically, in a squamous cell carcinoma model, deletion of non-malignant CD4 T cells decreased neoplastic cell progression and tumor incidence [114] underscoring the intimate relationship between inflammation and cancer. In CTCL, the indirect mechanism by which bacterial superantigens activate otherwise non-specific or TCR-deficient malignant T cells predicts that bacterial infections promote expansion of malignant T cells in an inflammatory setting with non-malignant T cells. In contrast, this model does not imply (but does not exclude) that bacterial superantigens have an etiological role in CTCL but does suggest a critical role of superantigens in the progression of CTCL. In keeping, it substantiates the general assumption that in progressive disease, malignant T cells in conjunction with the tumor environment are driven towards promoting a diverted inflammatory response, which boosts malignant T cell growth, exacerbates disease and likely increases susceptibility to additional infection.

## 8. Clinical Improvement after Antibiotic Treatment

Because many patients are suffering from recurrent bacterial infections, clinicians are often reluctant to undertake an aggressive treatment of skin infections with antibiotics due to the risk of increasing resistance to antibiotics. However, important clinical findings lend support to the hypothesis, that bacterial infections complicate and promote disease progression in CTCL. In several small series and case studies, elimination of *S. aureus* infection with antibiotics was associated with a rapid clinical improvement: in some patients treatment resulted in complete clinical response with no residual skin involvement by CTCL [83,115,116]. In an early study by Tokura and colleagues, clinical improvement in skin disease was observed after treating two CTCL patients with antibacterial agents [116]. In addition, Duvic and co-workers reported that in patients infected with SE-producing *S. aureus*, treatment with antibiotics resulted in clear clinical improvement [83]. Another study [115] reported on a high degree of *S. aureus* colonization in CTCL patients with an increased incidence in advanced erythrodermic SS patients when compared to non-erythrodermic MF patients. Eradication of *S. aureus* from the nostrils with oral and topical antibiotics was achieved in 85% of cases and similar treatment of skin lesions was effective in 91%. Consequently, after 4–8 weeks significant clinical improvement was seen in a majority of the treated patients [115]. Collectively, these observations speak in favor of an aggressive antibiotic treatment of bacterial infections in CTCL patients. Moreover, they are in support of the mechanism proposed above that bacterial toxins promote CTCL disease progression in CTCL. 

## 9. Conclusions

In conclusion, bacterial infections are a major clinical problem in CTCL and an important driver of morbidity and mortality in this disease. Despite much effort, definitive evidence supporting a direct etiological role of bacterial toxins is still lacking but other evidence suggests that toxins may also promote malignant T-cell expansion through a mechanism involving cross-talk between the malignant and non-malignant T cells. Given the proposed model for toxins as drivers of disease progression and the promising clinical data showing a beneficial effect of antibiotics on both morbidity and disease progression, we propose that an aggressive strategy for anti-bacterial therapy should be considered in all patients with clinically relevant and verified infections with *S. aureus*. 
